# Nasopharyngeal Microbiome Differences Between Children with Hypertrophic Adenoids and Healthy Controls

**DOI:** 10.3390/biology15151219

**Published:** 2026-07-23

**Authors:** Ulrich Moser, Thomas Hirsch, Beate Obermüller, Georg Singer, Vanessa Wolfschluckner, Dietmar Thurnher, Alexandros Andrianakis

**Affiliations:** 1 Department of Otorhinolaryngology, Medical University of Graz, 8010 Graz, Austria; 2 Department of Pediatric and Adolescent Surgery, Medical University of Graz, 8010 Graz, Austria

**Keywords:** nasopharyngeal microbiome, adenoidal microbiome, adenoids, adenoid hypertrophy, 16s rRNA gene sequencing, commensal microbiome, dysbiosis

## Abstract

Background: Adenoids play a pivotal role in immune system maturation during early childhood; however, pathological adenoid hypertrophy is associated with significant morbidity. Although infectious and immunological factors have been implicated, the role of the nasopharyngeal microbiome in adenoid hypertrophy remains incompletely understood. Methods: In this cross-sectional study, the nasopharyngeal microbiome of 26 children diagnosed with symptomatic hypertrophic adenoids was compared with that of 30 age-matched healthy controls with non-hypertrophic adenoids. Microbial profiling was performed using 16S rRNA (ribosomal ribonucleic acid)gene sequencing. Alpha and beta diversity, core microbiome, network and differential abundance analyses were performed. Results: Alpha diversity showed significant group differences (Fisher’s alpha *p* = 0.048), while beta diversity was not significant. Both groups had a common core microbiome dominated by *Moraxella*, *Haemophilus*, *Staphylococcus*, *Streptococcus*, *Corynebacterium* and *Dolosigranulum*, while *Fusobacterium* was identified exclusively in the study group. *Dolosigranulum*, *Corynebacterium*, *Finegoldia* and *Micrococcus* were characteristic in the control group, while *Campylobacter*, *Paludibacteraceae*, *Fusobacterium*, *Escherichia-Shigella*, *Cutibacterium* and *Nocardia* were typical in the study group. Conclusions: When compared with healthy controls, children with symptomatic hypertrophic adenoids exhibit a distinct nasopharyngeal microbiome characterized by altered microbial community structure and shifts in specific bacterial taxa. These findings support a potential role of the microbiome in the pathogenesis of adenoid hypertrophy.

## Simple Summary

Adenoid hypertrophy is a common childhood condition. However, the role of the nasopharyngeal microbiome in the pathogenesis of adenoid hypertrophy has not yet been fully elucidated. In this study, we examined the nasopharyngeal microbiome of children with symptomatic adenoid hypertrophy compared to healthy children with age-appropriate adenoid size. Our results show significant differences in the microbial community associated with hypertrophic adenoids, including the presence of inflammation-associated taxa such as *Fusobacterium*, *Campylobacter* and *Escherichia-Shigella*. In healthy children, by contrast, genera such as *Dolosigranulum*, *Corynebacterium*, *Finegoldia* and *Micrococcus* were characteristic. These findings suggest that microbiome dysbiosis may contribute to the pathogenesis of adenoid hypertrophy.

## 1. Introduction

Adenoids are conglomerates of lymphatic tissue located at the roof of the nasopharynx. Due to continuous exposure to continuously inhaled microorganisms, variations in oxygen content and humidity, and anatomical specificities, the nasopharynx forms a unique habitat for the resident microbiome [1]. Adenoids play an important role in early childhood by supporting the maturation of the adaptive immune system and the production of antibodies. This physiological process involves interaction with microbial antigens in the adenoids; consequently, a temporary enlargement of the adenoids during this period is normal and must be distinguished from pathological adenoid hypertrophy [2,3].

Hypertrophic adenoid disease has been associated with multi-microbial infections often of viral origin as well as with encapsulated bacteria and anaerobic, beta-lactamase-producing bacteria and individual host resistance [4,5]. The distinction between physiological and pathological enlargement of the adenoids is based on secondary symptoms such as mouth breathing, snoring, adenoidal facial expression, persistent secretory otitis media, recurrent acute otitis media, and chronic rhinitis or rhinosinusitis. The prevalence of pathological hypertrophic adenoids is approximately 34% in representative samples, although in various data sets the prevalence ranges from 42% to 70% [6]. Adenoid size depends on age, with the extent of hypertrophy being greater in children under 6 years of age than in older children [7]. A natural regression of the adenoids can be observed during puberty [8].

In early life, the nasopharyngeal microbiome is dominated by 6 bacterial genera—*Moraxella*, *Streptococcus*, *Staphylococcus*, *Haemophilus*, *Corynebacterium* and *Dolosigranulum*—which likely originate from maternal skin, vaginal and breast milk precursors [9,10]. Among these, *Streptococcus pneumoniae*, *Staphylococcus aureus*, *Haemophilus influenzae* and *Moraxella catarrhalis* are considered opportunistic pathogens, whereas *Dolosigranulum* and *Corynebacterium* are regarded as commensal genera [11]. The composition of the nasopharyngeal microbiome is influenced by multiple factors including immune status, socioeconomic conditions, season, age and bacterial interactions [12]. *Streptococcus pneumoniae* colonization is more common in children who regularly attend daycare, with rates of up to 50% for children aged 2 to 3, declining to around 10% in adults [13].

Numerous studies have already examined the microbiome of the adenoids, often in combination with that of the tonsils [14], and frequently in comparative analyses with chronic otitis media [15]. However, studies that explicitly compare the nasopharyngeal microbiome of healthy children with that of children with adenoid hypertrophy are rare [16]. There has been limited research on how the microbiome of the nasopharynx differs in pathologically enlarged adenoids compared to normal-sized adenoids. The aim of this study was to gain a deeper understanding of the nasopharyngeal microbiome and to identify potential associations with the occurrence of pathological adenoid hypertrophy. We hypothesized that the diversity and composition of the nasopharyngeal microbiome differ between children with pathological adenoid hypertrophy and age-matched healthy controls.

## 2. Materials and Methods

### 2.1. Study Design and Participants

This cross-sectional study was conducted at the Medical University of Graz between April and October 2019. This study was designed as an exploratory pilot study; therefore, no formal a priori calculation of the sample size was performed. A total of 60 children were consecutively enrolled: 30 with pathological hypertrophic adenoids and typical secondary symptoms (study group) and 30 healthy age-matched children with age-appropriate adenoid sizes and no pathognomonic symptoms (control group). Adenoidal mucus samples were collected from each participant during surgery under general anesthesia. Children in the study group were recruited at the Department of Otolaryngology, and healthy controls at the Department of Pediatric and Adolescent Surgery.

Inclusion criteria for the study group were:

- Planned adenoidectomy;
- Intraoperatively confirmed pathologic hypertrophic adenoids;
- Age between 2 and 10 years;
- At least three of the typical symptoms related to hypertrophic adenoids (trouble breathing through the nose, breathing through the mouth, adenoid facial expression, recurring rhinitis or rhinosinusitis, recurring otitis media and snoring/obstructive sleep apnea).

Children were eligible for the control group if they met the following criteria:

- Scheduled for non-infectious elective surgery unrelated to the adenoids (e.g., removal of implants following osteosynthesis for fractures);
- Had non-pathologic adenoids (no typical symptoms related to hypertrophic adenoids);
- Aged between 2 and 10 years.

Exclusion criteria for both groups were:

- Prior adenoidectomy;
- Acute upper respiratory tract infection;
- Systemic or topical antibiotics or corticosteroids within the preceding six weeks;
- Chronic respiratory diseases such as bronchial asthma or cystic fibrosis;
- Anatomical abnormalities of the nose or nasopharynx (e.g., nasal polyps or cleft palate).

### 2.2. Sample Collection and Processing

At the start of surgery, immediately after induction of general anesthesia, adenoidal mucus samples were collected with a disposable sinus secretion collector (Medtronic, Jacksonville, FL, USA) under endoscopic guidance. Samples were stored at −80 °C until further analysis. For DNA extraction, samples were thawed and dissolved in 500 μL of 0.9% NaCl. Microbial DNA was extracted using a MagNA Lyser device and the MagNA pure LC DNA Isolation Kit III (Roche Diagnostics, Mannheim, Germany) according to the manufacturer’s instructions. The microbial 16S rRNA gene was amplified with the universal primer pair 515FB and 806RB following the amplification protocol for bacterial community profiling [17]. The MyCycler^TM^ Thermal Cycler System and the T100^TM^ Thermal Cycler (Bio-Rad, Hercules, CA, USA) were utilized for amplification. The resulting fragments were analyzed and identified using a 3% agarose gel with the FastRuler Low Range DNA Ladder, ready-to-use, SM1103 (Thermo Fisher Scientific, Vilnius, Lithuania). Sequencing was then carried out using Illumina MiSeq (Illumnia, San Diego, CA, USA) at the Center for Medical Research (ZMF) at the Medical University of Graz.

### 2.3. Microbiome and Statistical Analysis

Raw sequencing data were processed using the QIIME2 platform. Sequence quality filtering, error correction, chimera removal, and inference of amplicon sequence variants (ASVs) were performed using the DADA2 denoising algorithm. The reads were assigned to classes by means of the SILVA 138 database for bacteria [18]. Parallel processed extraction blanks (negative controls) and contaminants caused by sample processing were removed using R Studio (version 12.1) and the R packages phyloseq, ggplot2, and decontam at a threshold of 0.5 [19]. Unaffiliated sequences, singletons and chloroplast-derived reads were also excluded. After quality filtering, a total of 3,558,977 reads were retained, with an average of 63,553 reads per sample (range: 8715–205,607 reads). Samples with fewer than 5000 reads were excluded to reduce the influence of low sequencing depth and biological background noise on diversity estimates and taxonomic profiling. The data were not rarified. Instead, prior to the subsequent analyses, normalization was performed using cumulative sum scaling (CSS), as this approach preserves the available sequencing information while accounting for differences in library size. Four patients of the study group were excluded due to insufficient sequencing depths of less than 5000 reads. This left 56 patients for the final analysis:, 26 in the study group and 30 in the control group. The resulting signatures were then exported to MicrobiomeAnalyst (version 2.0) for further processing [20]. Alpha diversity at the genus level was calculated using Fisher’s Alpha, the Shannon index and Chao1. Statistical analysis of the alpha diversity was performed with the Welch t-test. The Bray–Curtis index, Jaccard index, and Jensen–Shannon divergence were used to represent beta diversity at the genus level, and statistical significance was assessed using PERMANOVA (permutation multivariate analysis of variance). Genera were considered part of the core microbiome if they reached a minimum relative abundance of 0.01% and were detected in at least 20% of all samples. Correlation network analysis was carried out by means of Spearman’s rank correlation coefficient to evaluate pairwise associations between bacterial genera based on relative abundance data. To identify taxa that significantly contributed to group differences, differential abundance analysis was performed using DESeq2 (Differential Expression analysis for Sequencing data 2), a method for testing the differential expression of genes or taxa with negative binomial generalized linear models. Due to the large dataset and the high number of tests, *p*-values were adjusted by means of the Benjamini–Hochberg method to control the false positive rate. LEfSe (Linear discriminant analysis effect size) analysis was used as a complementary, exploratory approach to identify taxa with potential biological relevance. Random Forest classification was conducted using 1000 trees and *mtry* = 7 predictors considered at each split with random feature selection enabled. Model performance was evaluated using the internal out-of-bag (OOB) error estimate. No additional hyperparameter optimization was performed. The two classes were balanced (control group: *n* = 30; study group: *n* = 26), and no class weighting or resampling was applied. A random forest importance analysis was performed to identify the bacterial genera that contributed to the distinction between the study and the control group based on the mean decrease in accuracy.

The statistical software IBM SPSS Statistics, Version 28 (IBM Corp., Armonk, NY, USA), was used for statistical analysis of demographic data. While continuous variables are presented as mean values ± standard deviations, categorical variables are shown as absolute numbers and percentages. The Pearson chi-square test was used to compare unpaired categorical variables. The data were tested for normal distribution using the Shapiro–Wilk test. The independent t-test was used to compare continuous parameters between the two groups. The statistical significance level was set at a two-sided *p*-value of <0.05.

## 3. Results

### 3.1. Participant Characteristics

A total of 26 children with pathologically hypertrophic adenoids and secondary symptoms and 30 healthy children with age-appropriate adenoids without corresponding symptoms were included. There were no statistically significant differences between the two groups in terms of age or sex (Table 1).

### 3.2. Microbial Diversity

**Figure 1:**
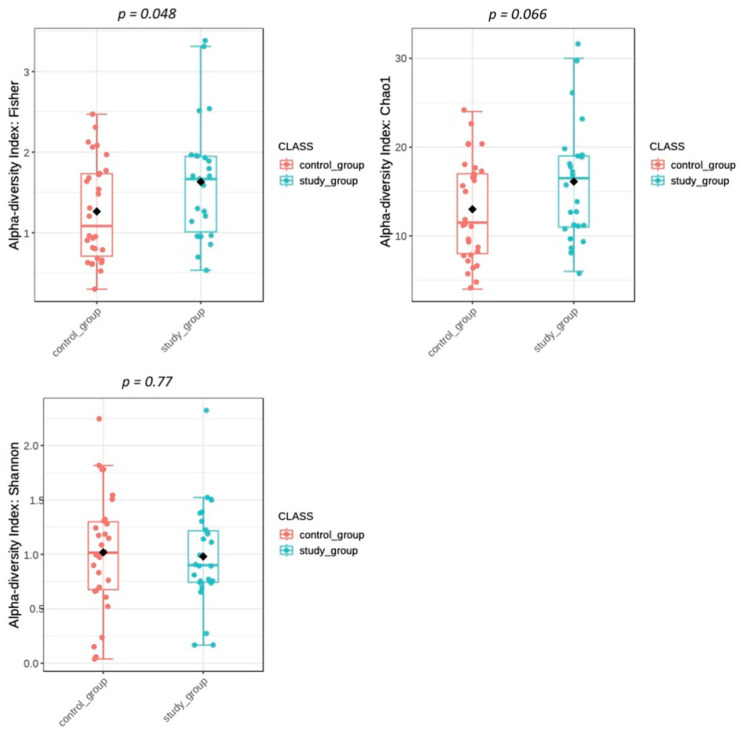
Alpha diversity at the genus level of the adenoidal microbiome of control and study group calculated using Fisher’s Alpha (*p* = 0.048), Chao1 (*p* = 0.066) and Shannon index (*p* = 0.77). These results suggest that differences between groups are due to shifts in rare genera rather than major changes in the community’s overall evenness. Statistical analysis was performed using the Welch *t*-test.

**Figure 2:**
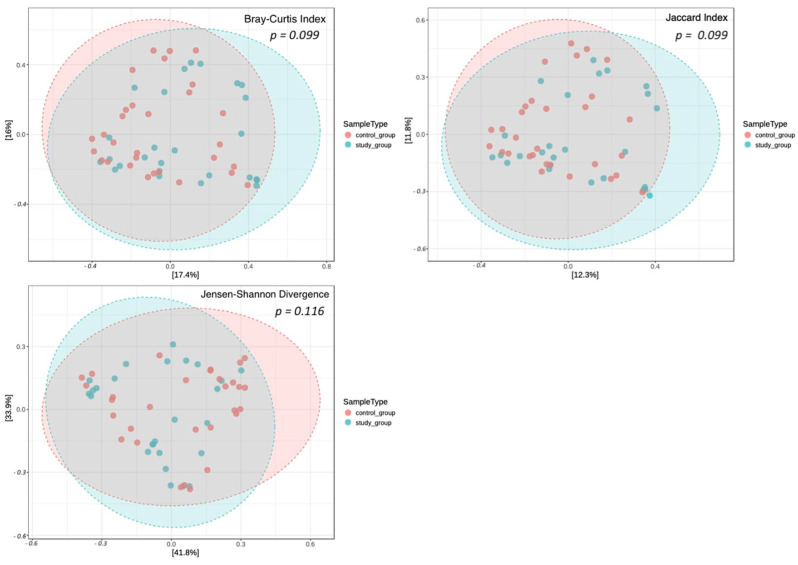
Beta diversity at the genus level of the adenoidal microbiome calculated using Bray–Curtis Index (*p* = 0.099), Jaccard index (*p* = 0.099) and Jensen–Shannon Divergence (*p* = 0.116). The shaded ellipses represent the 95% confidence intervals around the group centroids. Considerable overlap between the ellipses of the control and study groups was observed for all three distance metrics, indicating a largely similar overall microbial community composition between groups. Statistical analysis was performed using PERMANOVA.

The Fisher alpha index revealed a significant difference in alpha diversity at the genus level (*p* = 0.048). In contrast, the Chao1 Index exhibited a non-significant trend (*p* = 0.066), and the Shannon index was not significantly different between the two groups (*p* = 0.77) (Figure 1). The analysis of beta diversity revealed no significant difference in the microbiome of the nasopharyngeal cavity depending on adenoid status in the Bray–Curtis index (*p* = 0.099), Jaccard index (*p* = 0.099), and Jensen–Shannon divergence (*p* = 0.116) (Figure 2).

### 3.3. Abundance of Dominant Bacterial Genera and Core Microbiome

**Figure 3:**
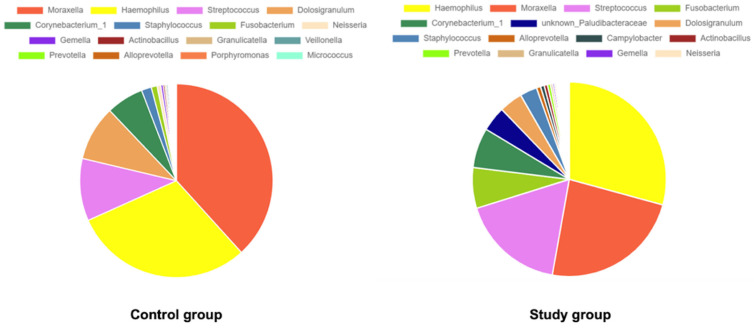
Pie chart showing the frequency of taxa at the genus level in the control and test groups, arranged in descending order.

When examining abundance, the genus *Moraxella* was most prevalent in the control group, followed by *Haemophilus*, *Streptococcus*, *Dolosigranulum*, *Corynebacterium*, *Staphylococcus*, *Fusobacterium* and *Neisseria*. In contrast, the most common bacterial genus in the study group was *Haemophilus*, followed by *Moraxella*, *Streptococcus*, *Fusobacterium*, *Corynebacterium*, *Paludibacteraceae*, and *Dolosigranulum* (Figure 3).

**Figure 4:**
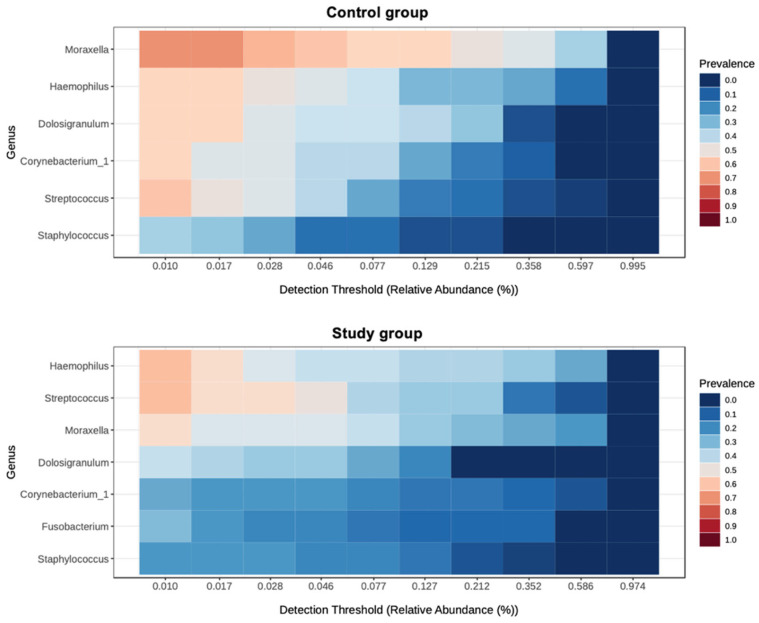
Prevalence heat maps of the core microbiome genera in the control group (**top**) and the study group (**bottom**). The majority of core genera were shared between groups, indicating a largely conserved core microbiome. Notably, Fusobacterium was identified as a core genus only in the study group, whereas it did not meet the predefined core microbiome criteria in the control group.

A comparison of the core microbiomes revealed a similar core composition in both groups. The following genera were present in both groups: *Staphylococcus*, *Streptococcus*, *Corynebacterium*, *Dolosigranulum*, *Haemophilus* and *Moraxella*. Notably, *Fusobacterium* was identified exclusively in the study group. Despite this shift, *Corynebacterium* and *Dolosigranulum* remained prevalent in both groups, albeit more so in the control group (Figure 4).

### 3.4. Network Analysis and Group-Specific Microbial Differences

**Figure 5:**
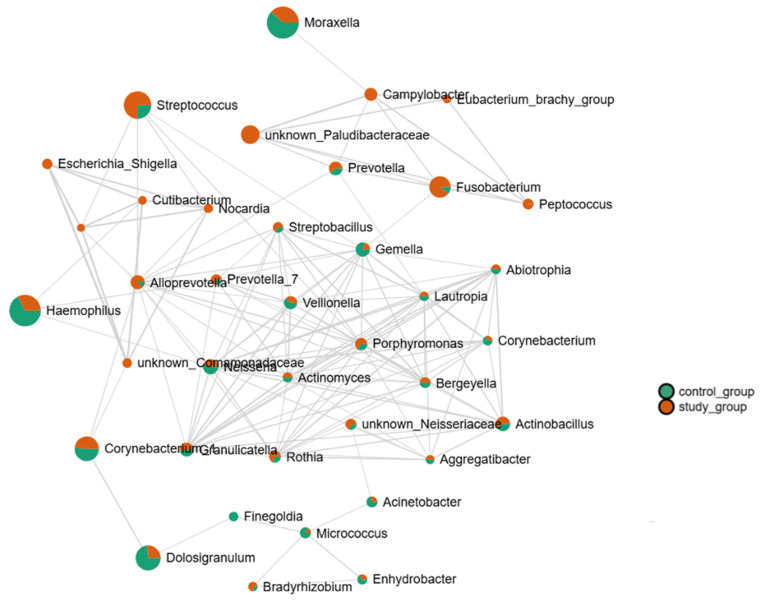
Correlation network of the adenoid microbiome at the genus level based on Spearman’s rank correlation coefficient. The nodes represent bacterial genera, with node size reflecting relative abundance and colors indicating group distribution (control group = green, study group = orange); the edges indicate significant correlations between genera. The network analysis revealed distinct co-occurrence patterns between groups, with genera such as *Dolosigranulum*, *Corynebacterium*, *Finegoldia*, and *Micrococcus* showing prominent interactions in the control group, whereas *Fusobacterium*, *Campylobacter*, *Paludibacteraceae*, *Streptococcus*, *Escherichia-Shigella*, *Cutibacterium* and *Nocardia* were more strongly connected within the study group network.

Correlation network analysis revealed distinct microbial clusters. The control group was characterized by a network dominated by *Dolosigranulum*, *Corynebacterium*, *Finegoldia* and *Micrococcus*. In contrast, the study group showed increased connectivity between *Fusobacterium*, *Campylobacter*, *Paludibacteraceae*, *Streptococcus*, *Escherichia-Shigella*, *Cutibacterium* and *Nocardia*. *Moraxella* was observed within the network associated with the study group, but without a central position, suggesting peripheral involvement rather than a key role (Figure 5).

**Figure 6:**
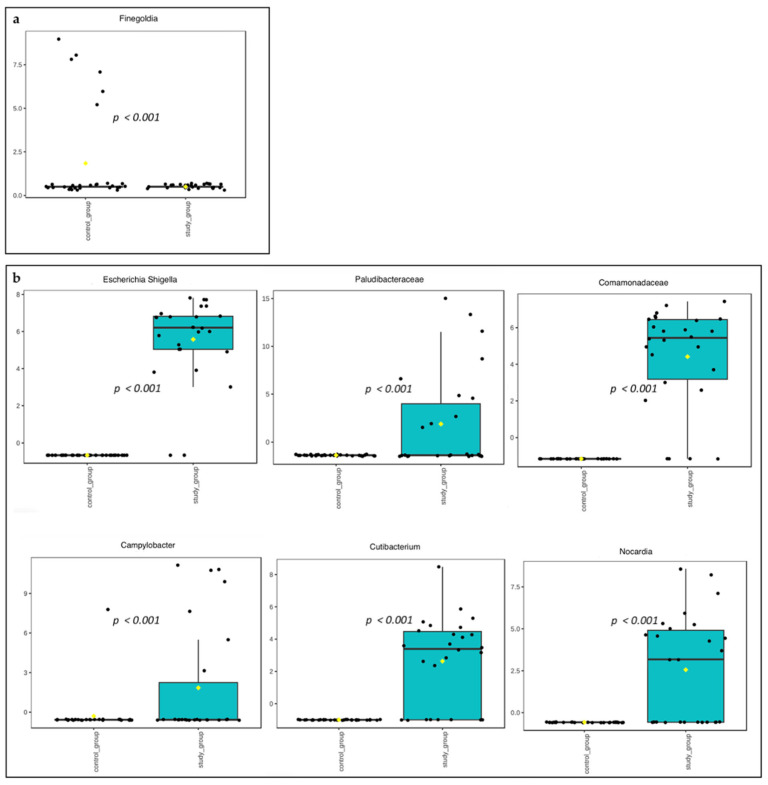
DESeq2-normalized counts of genera showing significant differences in relative abundance between the control and study groups. Panel (**a**) displays genera with higher abundance in the control group, whereas panel (**b**) displays genera with higher abundance in the study group. Differentially abundant taxa were identified using DESeq2 analysis. Black dots represent individual samples. The box plots show the distribution of DESeq2-normalized counts, with the center line indicating the median and the box representing the interquartile range. Yellow diamonds indicate the mean frequency. The *p*-values refer to the statistical comparison between the control group and the study groups.

A DESeq2 analysis was performed to detect taxa that contributed significantly to the differences between the groups. *Finegoldia* (*p* < 0.001) was significantly more prevalent in the control group (Figure 6a). Conversely, the study group was characterized by *Escherichia-Shigella* (*p* < 0.001), *Paludibacteraceae* (*p* < 0.001), *Comamonadaceae* (*p* < 0.001), *Campylobacter* (*p* < 0.001), *Cutibacterium* (*p* < 0.001) and *Nocardia* (*p* < 0.001). Figure 6b shows the box plots of the DESeq2 analysis for the study group. These results are in accordance with the LEfSe analysis: In the control group, *Finegoldia* was found to be present at a statistically significant level (*p* = 0.01), whereas the study group showed significant levels of *Escherichia-Shigella* (*p* < 0.001), *Comamonadaceae* (*p* < 0.001), *Cutibacterium* (*p* < 0.001), *Nocardia* (*p* < 0.001), *Paludibacteraceae* (*p* < 0.001) and *Campylobacter* (*p* = 0.01).

**Figure 7:**
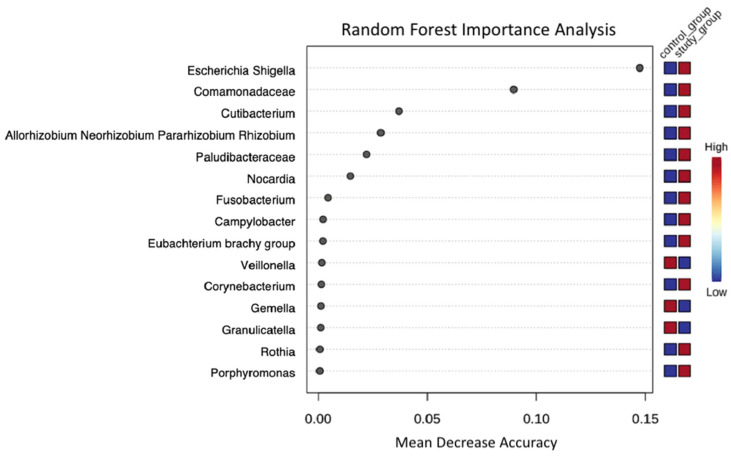
Random forest importance analysis showing the significance of the features that contribute to the distinction between the control and study groups. The significance is expressed as the average decrease in accuracy, with higher values indicating a stronger influence on classification accuracy. *Escherichia-Shigella*, *Comamonadaceae*, and *Cutibacterium* were the most influential characteristics for group discrimination.

Random forest classification demonstrated significant discriminatory power between the control and study group, achieving an OOB of 5.36%. All control samples were correctly classified, while three samples from the study group were misclassified, suggesting slightly higher heterogeneity in the study group. The random forest feature importance analysis identified several bacterial genera that contributed significantly to group discrimination. These included *Escherichia-Shigella*, *Comamonadaceae*, *Cutibacterium*, *Paludibacteraceae*, *Nocardia* and *Fusobacterium*, all of which were associated with pathological hypertrophic adenoids. *Veillonella* and *Gemella*, on the other hand, were assigned to the control group (Figure 7).

## 4. Discussion

In this study, we compared the nasopharyngeal microbiome of children with symptomatic hypertrophic adenoids to that of healthy children with age-appropriate non-hypertrophic adenoids. The observed significant differences in alpha diversity (*p* = 0.048) for Fisher’s alpha index, as well as the absence of significant differences in beta diversity, suggest that the overall composition of the microbiota remained largely stable in both groups and that the observed differences are likely due to changes in the relative abundance of certain taxa rather than to a complete reorganization of the adenoid microbiome.

The distinction between diseased and healthy children—and not the relationship between the microbiome and adenoid size—was relevant to our study. Therefore, the adenoids were not classified precisely by size (grades 1–4) [21], but were instead assigned to the study or control group based on the presence or absence of typical adenoid symptoms.

*Fusobacterium* was identified exclusively in the core microbiome of the study group, suggesting a shift toward anaerobic and potentially inflammation-associated taxa. It has been observed that *Fusobacterium* exhibits conditional duality: under physiological circumstances it may act as a harmless commensal, but under dysbiotic conditions, it may exhibit pathogenic behavior [22]. Brook et al. found that Fusobacterium was more common in children diagnosed with pathological hypertrophic adenoids than in children classified as healthy [23]. *Dolosigranulum* and *Corynebacterium* remained prevalent in the core microbiome of both groups, albeit with a higher abundance in the control group. However, the fact that both remained in the study group indicates that their physiological core microbiome had been partially preserved. Previous reports have claimed that higher rates of *Dolosigranulum* and *Corynebacterium* act protectively against respiratory pathogens and contribute to a stable nasopharyngeal microbiome profile [24,25,26]. Similarly, stable microbiome profiles in the gut have been associated with health, whereas an unstable microbiome may predispose to disease [27,28].

This result was consistent with our network analysis findings, which showed that the control group had networks dominated by genera associated with microbial stability and respiratory health, such as *Dolosigranulum*, *Corynebacterium*, *Finegoldia* and *Micrococcus*. To substantiate these results, *Finegoldia* was detected as significantly relevant for the control group in the differential analysis (*p* < 0.001). *Finegoldia* is classified as an anaerobic bacterium that colonizes the oral cavity, among other sites, and does not cause inflammation in healthy hosts. However, it can become an opportunistic pathogen in wounds or in cases of immune deficiency [29,30]. When *Micrococcus* colonizes the nasopharynx, a downward trend in the frequency of acute middle ear infections has been observed [31].

Network analysis revealed additional disparities in the study group, with elevated levels of connectivity between more anaerobic and inflammation-associated genera, including *Campylobacter*, *Paludibacteraceae*, *Streptococcus*, *Fusobacterium*, *Escherichia-Shigella*, *Cutibacterium* and *Nocardia*. These results were substantiated by the detection of *Campylobacter* (*p* < 0.001), *Paludibacteraceae* (*p* < 0.001), *Escherichia-Shigella* (*p* < 0.001) *Cutibacterium* (*p* < 0.001) and *Nocardia* (*p* < 0.001) in the differential analyses. The increased co-occurrence and connectivity of these elements suggest the presence of cooperative microbial networks that may be associated with persistent inflammatory conditions and may contribute to adenoid hypertrophy. Previous reports have shown increased *Cutibacterium* abundance in the nasopharynx of children with chronic sinusitis [32] and during COVID-19 infection [33]. In a comparative study of the upper respiratory tract microbiome in children with obstructive sleep apnea and hypertrophic adenoids, Zhang et al. found an increased relative abundance of *Fusobacterium* and *Campylobacter* in the adenoidal microbiome of the study group [34]. A recent study on rhinosinusitis in children demonstrated that *Fusobacterium* and *Campylobacter* can be detected more frequently in nasopharyngeal samples in chronic disease [35]. *Escherichia-Shigella*, and *Nocardia* are not commonly found in the pharynx. *Escherichia-Shigella* is typically part of the intestinal microbiome [36]. *Nocardia* is a bacterium that is ubiquitous in the environment and is responsible for localized or disseminated infections in humans [37]. While the detection of *Fusobacterium*, *Campylobacter*, *Escherichia-Shigella*, and *Nocardia* could indicate actual colonization of hypertrophic adenoids, it could also be due to temporary environmental contamination or low-abundance signals of uncertain biological relevance. Given their low prevalence and the cross-sectional nature of this study, the ecological and clinical relevance and causality of these taxa remain uncertain. Therefore, their association with hypertrophic adenoids should be interpreted with caution.

In the present study, *Moraxella* reflected the inflammatory microbial environment of hypertrophic adenoids rather than acting as a primary driver of the disease-associated network architecture. Based on existing data, the carrier rate of *Moraxella catarrhalis* in the nasopharynx ranges between 22% and 70% [38,39]. Biesbroek et al. observed a lower rate of upper respiratory tract infections in children with *Moraxella*-dominant microbiome compositions [40]. Recent findings even indicate that *Moraxella* acts largely as a bystander in infections rather than as a true pathogenic agent [41,42,43].

The recently published study by Sokolovs-Karijs, which reports similar microbial patterns in children with hypertrophic adenoids, may suggest that these changes in the nasopharyngeal microbiome are reproducible across different populations [16]. In the aforementioned study, samples for the control group were collected via nasopharyngeal swabs exclusively from 5- to 7-year-old children who were conscious—a procedure that can be significantly more difficult or prone to error when dealing with an awake, uncooperative child. In contrast, all the children in the control group of our study were under anesthesia, which ensured a proper sample collection. In addition, special disposable secretion collectors were used, as they could scrub the surface and aspirate into the crypts of the adenoids.

The following factors were considered as potential confounders on the nasopharyngeal microbiome in our study: age, mode of delivery, previous upper respiratory tract infections, chronic respiratory diseases, and recent use of corticosteroids. However, attendance at a daycare center and exposure to passive smoke were not considered as potential confounding factors in our study and may therefore have contributed to possible spurious changes in the nasopharyngeal microbiome.

The observed differences in the microbial composition between the study and control groups may contribute to the development or prevention of pathologically enlarged adenoids. Although attributing the disease solely to changes in the bacterial microbiome would be too simplistic, our findings provide new insights into the possible microbial mechanisms that trigger adenoid pathology. In the future, microbiome-based diagnostics could help identify children at risk of developing hypertrophic adenoids. Furthermore, a deeper understanding of the dynamics of microbial communities could facilitate the development of targeted interventions that modulate the nasopharyngeal microbiome. It should be noted, however, that such applications are currently speculative and must be validated in larger longitudinal and mechanistic studies.

### Limitations

This study has several limitations. The sample size was relatively small, which may limit statistical power and generalizability of the results. Moreover, only a single sample per participant was analyzed, preventing assessment of temporal or longitudinal changes in microbial composition. Furthermore, the cross-sectional design precludes conclusions regarding causality between microbial alterations and adenoid hypertrophy. The study focused exclusively on bacterial 16S rRNA sequencing and therefore did not assess viral, fungal or functional microbial components that may also contribute to disease pathogenesis. Finally, although extensive decontamination procedures were applied, low-biomass respiratory microbiome studies remain susceptible to residual environmental contamination, requiring cautious interpretation of uncommon taxa.

## 5. Conclusions

This study provides new insights into the composition of the nasopharyngeal microbiome in children with symptomatic hypertrophic adenoids compared with healthy children. The results suggest subtle but detectable differences in microbial composition on adenoid hypertrophy. Further studies involving larger cohorts and longitudinal sampling are needed to clarify causal relationships and to investigate microbiome-targeted interventions, such as probiotics, as potential preventive or complementary therapeutic strategies for hypertrophic adenoid disease in children.

## Figures and Tables

**Table 1:** Demographic data of the study and the control group.

Parameter	Whole Cohort (*n* = 56)	Control Group (*n* = 30)	Study Group (*n* = 26)	*p*-Value
Age, years	5.2 ± 2.1	5.0 ± 2.1	5.4 ± 2.1	0.410
Sex				0.472
Females, no. (%)	23 (41%)	11 (37%)	12 (47%)	
Males, no (%)	33 (59%)	19 (63%)	14 (53%)	
Birth mode				0.235
Natural, no. (%)	41 (73%)	20 (67%)	21 (81%)	
Cesarean section, no (%)	15 (27%)	10 (33%)	5 (19%)	
Allergy	4 (7.14%)	1 (3.33%)	3 (11.54%)	0.328

Continuous variables are presented as means ± standard deviations and categorical variables as absolute numbers and percentages.

## Data Availability

The datasets generated and analyzed during the current study are available from the corresponding author upon reasonable request due to ethical restrictions and university regulations.
